# Draft genome sequences of three rhodopsin possessing *Croceitalea* sp. strains, isolated from the sea surface microlayer in Japan

**DOI:** 10.1128/mra.00038-24

**Published:** 2024-02-16

**Authors:** Mako Takada, Chunqi Jiang, Youta Sugai, Masumi Hasegawa-Takano, Takayoshi Fujiwara, Yuya Tsukamoto, Yu Nakajima, Yosuke Nishimura, Susumu Yoshizawa

**Affiliations:** 1 Atmosphere and Ocean Research Institute, The University of Tokyo, Kashiwa, Chiba, Japan; 2 Department of Natural Environmental Studies, Graduate School of Frontier Sciences, The University of Tokyo, Kashiwa, Chiba, Japan; 3 Institute for Extra-Cutting-Edge Science and Technology Avant-Garde Research (X-star), Japan Agency for Marine-Earth Science and Technology, Yokosuka, Kanagawa, Japan; 4 Research Center for Bioscience and Nanoscience (CeBN), Research Institute for Marine Resources Utilization, Japan Agency for Marine-Earth Science and Technology (JAMSTEC), Yokosuka, Kanagawa, Japan; Montana State University, USA

**Keywords:** *Flavobacteriaceae*, Croceitalea, rhodopsin, ocean

## Abstract

Here, we present the draft genome sequences of three *Croceitalea* sp. strains containing microbial rhodopsins, isolated from the Japanese coastal sea surface microlayer, which is exposed to intense sunlight. This study will contribute to the understanding of the genus *Croceitalea* and the diversity of microbial rhodopsins.

## ANNOUNCEMENT

The genus *Croceitalea* belongs to the family *Flavobacteriaceae* in the phylum *Bacteroidota*. To date, four type species have been described in this genus, and members of this genus are characterized by Gram-negative, rod-shaped, yellow-orange colonies (1–3). Light-activated, ion-pumping rhodopsins are widely distributed among various bacteria and archaea which inhabit the photic zone of aquatic environments (4). Marine flavobacteria have been consistently found to possess rhodopsin genes, suggesting their ability to harness sunlight energy (5).

Strains used in this study were isolated from the sea surface microlayer (SML) of coastal Sagami Bay, Japan (35°09′04″N, 139°08′47″E). SML samples were collected by placing 47 mm polycarbonate membrane filters (Whatman, United Kingdom) onto the water surface for 10 s and spread onto 1/10-strength ZoBell agar plates (6–8). The plates were irradiated with UV-C (254 nm) at 595 μW/cm^2^ for 60 s using a handy UV lamp (UVGL-25, Funakoshi, Japan) and incubated at 25°C for 2 weeks under white light condition. After incubation, the colonies were re-isolated onto 1/2 ZoBell agar plates, and three isolates (strains MTPC5, MTPC6, and MTPC9) were obtained. They were incubated in 10 mL of 1/10 ZoBell medium at 25°C for 1 week for DNA extraction. Genomic DNA samples were extracted using Wizard Genomic DNA puriﬁcation kit (Promega, USA). Library preparation was performed with MGIEasy PCR-Free DNA Library PreSet and paired-end sequencing (2 × 150 bp) was performed with DNBSEQ-G400 Genetic Sequencer (MGI Tech, China) by Genome-Lead Ltd. (Kagawa, Japan). In total, 10,165,864 (MTPC5), 11,172,170 (MTPC6), and 12,989,703 (MTPC9) paired-end reads were obtained. The obtained reads were checked using FastQC v 0.12.0 (9) and trimmed using fastp v0.22.0 (10). The trimmed reads were assembled using Unicycler v0.4.8 (11). The assemblies were annotated using DDBJ Fast Annotation and Submission Tool (DFAST) v1.2.0 with the “Prodigal” and “tRNAscan-SE (Bacteria)” parameters (12). The qualities of the assemblies were checked using CheckM v1.2.2 (13). Average nucleotide identity (ANI) was calculated using the Genome-based distance matrix calculator (14). Digital DNA-DNA hybridization (dDDH) was calculated using the Genome-to-Genome Distance Calculator 3.0 (15).

The 16S rRNA gene sequence similarity values indicated that they belong to the genus *Croceitalea*, with the closest relatives being *Croceitalea eckloniae* DOKDO 025^T^ (MTPC5, 97.80%), *Croceitalea marina* H01-35^T^ (MTPC6, 96.84%), and *C. marina* H01-35^T^ (MTPC9, 96.84%), respectively. The ANI and dDDH values calculated using three genome sequences determined in this study consistently revealed 75.59%–77.35% ANI values and 17.4%–18.3% dDDH values against the public genomes of closely related strains: *C. dokdonensis* DOKDO 023^T^ (ASM130641v1), *Croceitalea* sp. F388 (ASM3184644v1), *Croceitalea* sp. P007 (ASM3184640v1), and *Croceitalea* sp. P059 (ASM3184636v1). MTPC6 and MTPC9 exhibited 100% similarity of both ANI and dDDH values, indicating they are likely the same species.

General genomic characteristics of these strains are listed in Table 1. Annotation results showed that these genomes share DNA repair and antioxidant genes, such as *phr* and *sod* which protect biomolecules from UV rays and oxidation (16, 17). Notably, each genome harbors a proteorhodopsin gene. This suggests that all three strains may have a photoheterotrophic lifestyle in the marine environment.

**TABLE 1:** Genome features of *Croceitalea* sp. strains MTPC5, MTPC6, and MTPC9

Genetic element	MTPC5	MTPC6	MTPC9
Number of contigs	55	54	60
Genome size (bp)	4,487,984	3,793,990	3,794,837
G + C content (%)	40.1	36.6	36.6
Coding sequences	4,061	3,551	3,545
tRNAs	37	39	39
rRNAs	3	3	3
Length of 16S rRNA (bp)	1,519	1,521	1,521
Genome coverage	339	440	512
N50	628,601	314,404	848,979
Completeness (%)	99.01	99.01	99.01
Contamination (%)	0.33	0.72	0.72
Accession numbers	BTGQ01000001-BTGQ01000055	BTGR01000001- BTGR01000054	BTGT01000001- BTGT01000060

## Data Availability

The draft genome sequences have been deposited in DDBJ/EMBL/GenBank under the accession numbers listed in Table 1. The genome sequencing raw reads have been deposited in the NCBI Sequence Read Archive under accession numbers DRR492745, DRR492746, and DRR492748, respectively.
